# SteganoCNN: Image Steganography with Generalization Ability Based on Convolutional Neural Network

**DOI:** 10.3390/e22101140

**Published:** 2020-10-08

**Authors:** Xintao Duan, Nao Liu, Mengxiao Gou, Wenxin Wang, Chuan Qin

**Affiliations:** 1 College of Computer and Information Engineering, Henan Normal University, Xinxiang 453007, China; liunao18437982698@126.com (N.L.); goumengxiao@126.com (M.G.); wangwenxin3023@126.com (W.W.); 2 School of Optical-Electrical and Computer Engineering, University of Shanghai for Science and Technology, Shanghai 200093, China; qin@usst.edu.cn

**Keywords:** image steganography, SteganoCNN, Unet, fully convolutional densely connected network (FCDenseNet)

## Abstract

Image-to-image steganography is hiding one image in another image. However, hiding two secret images into one carrier image is a challenge today. The application of image steganography based on deep learning in real-life is relatively rare. In this paper, a new Steganography Convolution Neural Network (SteganoCNN) model is proposed, which solves the problem of two images embedded in a carrier image and can effectively reconstruct two secret images. SteganoCNN has two modules, an encoding network, and a decoding network, whereas the decoding network includes two extraction networks. First, the entire network is trained end-to-end, the encoding network automatically embeds the secret image into the carrier image, and the decoding network is used to reconstruct two different secret images. The experimental results show that the proposed steganography scheme has a maximum image payload capacity of 47.92 bits per pixel, and at the same time, it can effectively avoid the detection of steganalysis tools while keeping the stego-image undistorted. Meanwhile, StegaoCNN has good generalization capabilities and can realize the steganography of different data types, such as remote sensing images and aerial images.

## 1. Introduction

Privacy protection of communication between two parties has been a hot topic on the internet for a long time, and the privacy protection of communication involved [1] can be combined with information hiding, thereby achieving secure communication [2]. Image steganography [3] is an important part of information hiding. The sender hides the message in the carrier image to obtain a secret image (stego), and sends this to the receiver. After receiving the stego-image, the receiver reconstructs the required message from it. Image is one of the main forms of information carrier, especially when it comes to sensitive information such as privacy and security. Its safe storage and transmission has always been a very important issue.

In recent years, with the unprecedented achievements of CNN in image segmentation [4] and image classification [5], the application of CNN to image steganography is also a hot topic today. The traditional image steganography proposed by Pevny et al. [6] has a maximum payload capacity of 0.4 bits per pixel. If it is greater than this point, the visual integrity of the human eye will be damaged. Xin et al. [7] embed information based on the complexity of the R, G, and B three-channel textures, which is different from the strategy of equally distributing secret information among the R, G, and B three channels. The maximum load rate of the carrier image is 0.3 bits per pixel. The continuous updating of computer hardware can effectively promote the development of deep learning and promote image steganography. Rahim et al. [8] first proposed an end-to-end decoding network encoding network architecture based on convolutional neural networks, which realized the scheme of hiding the secret image in the carrier image. Given the known stego-image and carrier image, our human visual system can easily distinguish the differences between the two images. The solution proposed by Ping Wu et al. [9] solves the distortion of the stego-image, thereby ensuring the visual integrity of the stego-image. Zhang et al. [10] decomposed the carrier image (color image) into three channels corresponding to the Y, U, and V channels. The secret image (gray image) is connected to the Y channel in series, and the output is similar to the carrier image after passing through the encoder. The Y channel is decomposed from the stego-image, and the secret image is extracted through the decoder. One advantage of this scheme is that a steganalyzer is added to the network, which realizes the adversarial between steganography and steganalysis, and has a certain ability to resist steganalysis.

In the information hiding system, the security, payload capacity and perception of the visual system can be used as evaluation indicators for image steganography. The security of image steganography depends on two factors: first, the amount of information to be hidden and second, the appearance of the carrier image itself and the amount of change in basic statistics after the message is embedded. Perfect image steganography is the maximum amount of hidden information, while the appearance of the stego-image and the basic statistical data remain unchanged. IThis is not feasible in practice, because the appearance of the carrier image and the amount of embedded information is a hostile relationship. The more embedded messages, the greater the degree of image distortion, and the lower the security of steganography, and vice versa.

Nowadays, the Generative Adversarial Network (GAN) [11] is developing rapidly, and methods of applying GAN to information hiding [12,13,14,15] are also emerging. Wang et al. [16] proposed using GAN for high-capacity information hiding, with a payload capacity of 0.4 bpp. A significant difference of HidingGAN is the addition of a steganalyzer for adversarial training, similar to the classic prisoner model problem. Zhou Zhang et al. [17] proposed a steganography scheme based on GAN and Cardan grid mask. First, the noise vector is used as the input of GAN for training, and the output is a natural image. The image is disturbed to obtain a disturbing image. Secondly, the secret information is embedded in the disturbing image, and the disturbing image is sent to the GAN to continue training and output as stego. This is sent to the recipient, who extracts the secret information through the Cardan grille mask agreed upon by both parties. The maximum relative load of this scheme is 1.49 × 10${}^{-3}$. A problem worth considering in this scheme is that the embedded secret information is different, but they must all be embedded in the same image. If they need to be embedded in different images, the network needs to be retrained.

**Figure 1:**
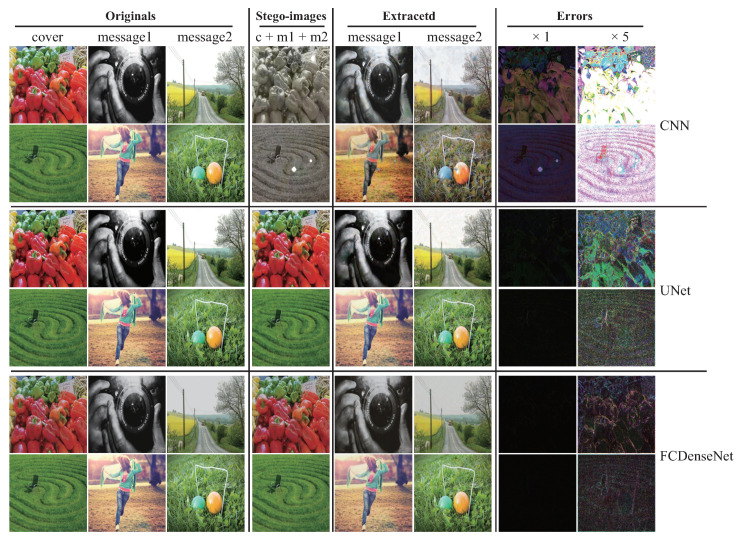
The sample example comes from SteganoCNN, an information hiding system that hides two secret images. Row: 1, 2, Row: 3, 4, Row: 5, 6 are from hidden networks ResNet, Unet and FCDenseNet, respectively. Columns 1 to 4 correspond to the original carrier image, secret image 1, secret image 2, and stego-image, and columns 5 to 8 correspond to the extracted secret image 1, secret image 2, error map ×1, ×10.

Aiming at the problem of the above-mentioned information-hiding scheme based on deep learning, how to effectively balance the security of image steganography and the amount of hidden information is a challenge in the current direction of information-hiding research. In this paper, we propose a steganography model (SteganoCNN) based on a deep convolutional neural network that can effectively increase the steganography capacity under the premise of ensuring steganography security. The experimental results are shown in Figure 1. Figure 1 corresponds to three different networks, respectively, CNN, Unet and FCDenseNet. The process of hiding and extracting is briefly described, as follows: First, the input of the encoder (hidden network) is a carrier image and two secret images. After the fusion of high-dimensional features, the encoder outputs the stego-image. Second, the input of the decoder is the stego-image. After processing by the decoder (two extraction sub-networks), two different secret images are output. There are two modules in SteganoCNN, the encoder and the decoder, which correspond to the hidden network and the extraction network. The encoder has two sub-modules corresponding to the two extraction sub-networks. The encoder automatically embeds two secret images into a carrier image to obtain a stego-image, and the decoder reconstructs two secret images from the stego-image.

The main contributions of this study are summarized as follows.

A Novel StegoCNN model is proposed by us. The network can automatically learn high-dimensional features of images and use them in a data-driven manner. It can effectively realize the fusion of high-dimensional features of the carrier image and the original secret image, so as to realize the embedding and extraction of secret images.

StegoCNN can realize high-capacity image steganography. The ratio of the secret image to be hidden in the carrier image is 2:1. At the same time, the payload capacity of the stego-image reaches 47.92 bit per pixel under the premise of ensuring visual integrity and anti-steganalysis.

The StegoCNN model has good generalization capabilities. We use images that are different from the ImagNet dataset, such as remote sensing images and aerial images, which can also effectively realize hiding and extraction under the premise of ensuring steganographic security.

The structure of this paper is organized as follows: Section 2 is related work; Section 3 will describe the proposed SteganoCNN model be proposed in detail; Section 4 will evaluate the SteganoCNN model through Experimental Analysis; the conclusion is presented in Section 5.

## 2. Related Work

Due to the rapid development of deep learning, a large number of academic researchers use deep-learning feature extraction to perform steganalysis and steganalysis of images, audio, video, and text. Chen et al. [18] proposed a two-channel image steganography (TDHN) scheme based on deep learning. First, the carrier image and the secret image are pooled and averaged separately, and then they are 1×1. After the convolution of the feature maps, the encrypted image is obtained after the Inception-Resnet module and 3×3 convolution. For the extraction of the secret image, the Inception-Resnet module is also used to obtain the secret image after 3×3 convolution.

Chen et al. [19] proposed a high-capacity robust image steganography method based on adversarial networks. The main idea comes from game theory. The sender tries to perfectly hide the secret information and send it to the receiver, while the listener tries to intercept the secret information from their communication. In the continuous game process, the two sides try their best to perfect their own shortcomings. The scheme proposed by Chen et al. [19] is to decompose the carrier image into RGB three channels, firstly performing convolution operation on the B channel and the secret image (gray image), and secondly, performing the concatenation operation to obtain the stego-image. The stego-image undergoes adversarial training with a steganalyzer. Finally, the secret image is obtained by extracting the network. Qin et al. [20] proposed a reversible image steganography scheme based on a GAN. This scheme uses a GAN for adversarial training. First, the secret information and the carrier image are used as the input of the encoder. The encoder outputs the stego-image. The output of the encoder is used as the input of the decoder, and the decoder extracts the secret information from the stego-image. In this scheme, the encoder and decoder use the same Dense Block structure, and the discriminator uses the Inception block structure. This scheme realizes reversible information steganography. Li et al. [21] proposed an image steganography scheme based on chaotic encryption and GAN. Increase the security of image steganography to a certain extent. First, perform a chaotic encryption operation on the secret image. Secondly, the encrypted secret image and carrier image is used as the input of the hidden network (Unet). The hidden network output is the stego-image. After the extraction network, the output is an encrypted secret image. After the chaos decryption algorithm, the secret image is extracted. The scheme then joins the discrimination network. The entire hidden network and the discrimination network are trained in an adversarial manner, which further enhances the effect of hiding. However, this scheme mainly aims to hide and extract grayscale images. Yu proposed [22] an image steganography model (ABDH) based on the attention module and GAN. First of all, the attention model mainly preprocesses the carrier image to obtain a high-texture area more suitable for secret image embedding. Secondly, the carrier image, the secret image, and the attention mask are used as the input of the generative model to obtain the stego-image. Finally, the extraction network extracts the secret image from the stego-image. Two adversarial pieces of training are added to ABDH. One is the adversarial training of the generative model and the discriminator. The other is to extract the adversarial training between the network and the entire model. The common point of the above schemes is to hide an image and extract an image. Although the hiding effect is very good, there are certain limitations to its capacity.

In our work, we first adjust the size of the image to 256 × 256, and then the carrier image is concatenation with two secret images, and the stego-image is obtained through the hidden network (the hidden network has three choices: CNN, Unet, and FCDenseNet) image; the input of the extraction network is stego-image, and the output is two secret images.

## 3. SteganoCNN Architecture

**Figure 2:**
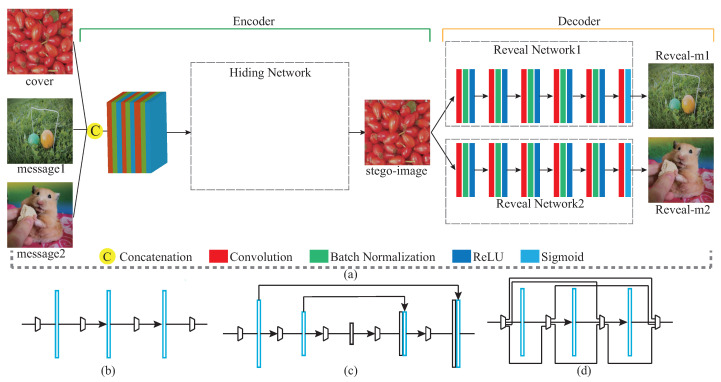
(**a**) is the framework of the entire SteganoCNN network. The hidden network part of the encoder module can be replaced by (**b**) or (**c**) or (**d**). (**b**–**d**) are the brief architectures of ordinary CNN, Unet, and FCDenseNert, respectively.

One common point in the schemes designed in [8,9,10,14,23] is that they use convolutional neural networks to embed only a secret image in the carrier image. The solution we designed is to embed two secret images into the carrier image and realize high-capacity steganography under the premise of ensuring steganography security. The framework of the entire model is shown in Figure 2. We are different from the solution [24]. In our SteganoCNN model, the preparation network is removed, and three different hidden networks ((b), (c), (d)) are used to train the entire network. Before training the network, we uniformly adjust the images input to the network to a size of 256 × 256. First, the carrier image and two secret images are concatenation on a channel, which can be described as $c\in R^{W\times H\times D}$, $s1\in R^{W\times H\times D^{\prime }}$ and $s2\in R^{W\times H\times D^{\prime \prime }}$ are carrier images, secret image 1 and secret image 2, and $\left(c,s1,s2\right)\to \phi \in R^{W\times H\times (D+D^{\prime }+D^{\prime \prime })}$ are carrier images and two secret images on the channel Series operation. Among them, *W*, *H* and *D* are the width, height and channel number of the picture, respectively. After the concatenation operation, a 9-channel feature map is obtained. This is sent to the hidden network to undergo a series of operations such as convolution, pooling and concatenation, and the output is a stego-image. Finally, the stego-image is sent to the extraction network and undergoes operations such as convolution and batch normalization to reconstruct two secret images. Our goal is to reduce the training error of the entire system as much as possible, realize the embedding of two secret images into one carrier image, and train the entire system by reducing the following errors:

(1) $$\zeta \left(c,c^{\prime },s1,s1^{\prime },s2,s2^{\prime }\right)=∥c-c^{\prime }∥+\beta \left(∥s1-s1^{\prime }∥+∥s2-s2^{\prime }∥\right)$$

Among them *c*, $c^{\prime }$, s1, $s1^{\prime }$, s2, $s2^{\prime }$ and β are the original carrier image, the secret image, the original secret image 1, the reconstructed secret image 1, the original secret image 2, the reconstructed secret image 2, and the weight of the network. $∥c-c^{\prime }∥$ is the training error of the hidden network in the encoder to optimize the parameters of the hidden network (reduce the error between the carrier image and the encrypted image), without changing the weights and parameters in the extraction network, because it does not need to reconstruct the secret image. $\beta (∥s1-s1^{\prime }∥+∥s2-s2^{\prime }∥)$ acts on the extraction network in the decoder (to reduce the error between the original secret and the reconstructed secret image), but it also affects the parameters and weights of the hidden network. The purpose of this is to better balance the carrier image and the stego-image, the secret image, and to reconstruct the quality of secrets.

To visualize the execution process of SteganoCNN hiding and extraction, we present it in pseudo-code, as follows.
**SteganoCNN Hidden and Extracted Procedures.****Input:***c*, s1, s2(1) Initialize the convolution operation weight: Normal(0.0,0.02);     Initialize the BatchNorm operation weight: Normal(1.0,0.02).(2)     x=Cat(c,s1,s2)(3)    **For**
*i* = 1 to 200 **do**(4)    $c^{\prime }=Encoder\left(x\right)$.(5)    Update the weight of the Encoder by $∥c-c^{\prime }∥$.(6)    $y=Decoder(c^{\prime })$.(7)    $s1^{\prime }=RevealNetwork1\left(y\right)$;        $s2^{\prime }=RevealNetwork2\left(y\right)$.(8)    Update the Encoder and Decoder weights through ζ.(9)    **End For****Output:**$c^{\prime }$, $s1^{\prime }$, $s2^{\prime }$

### 3.1. Encoder That Hides Secret Images

The input of the hidden network is a nine-channel feature map. The hidden network in the encoder module can be replaced by any of the following: (b) ordinary CNN, (c) Unet, (d) FCDenseNet in Figure 2. (b), (c), and (d) in Figure 2 are their schematic diagrams. y×yConv represents a convolution operation whose size of convolution kernel is y×y, z(…) and represents performing the same operation *z* times, where *y* and *z* are positive integers. The process represents the execution process of the hidden network, outputsize is the size of the output feature map expressed by W×H×D, where *W*, *H*, *D* correspond to the width, height, and channel number of the feature map, respectively. The main function of the encoder is to automatically embed secret information into the color bits and channels of the carrier image through the convolutional neural network.

#### 3.1.1. Ordinary CNN

The description of the detailed structure in Figure 2b is shown in Table 1. The first column indicates the order of execution, and the second column indicates the size of the output feature map.

#### 3.1.2. Unet

Unet is mainly used for medical image segmentation [25], 3D video segmentation [26], and has achieved good results in segmentation. Please refer to [23] for the detailed structure of (c) in Figure 2. All convolution kernels in this network have the same size of 4 × 4, with a step size of 2, and a padding of 1.

#### 3.1.3. FCDenseNet

**Figure 3:**
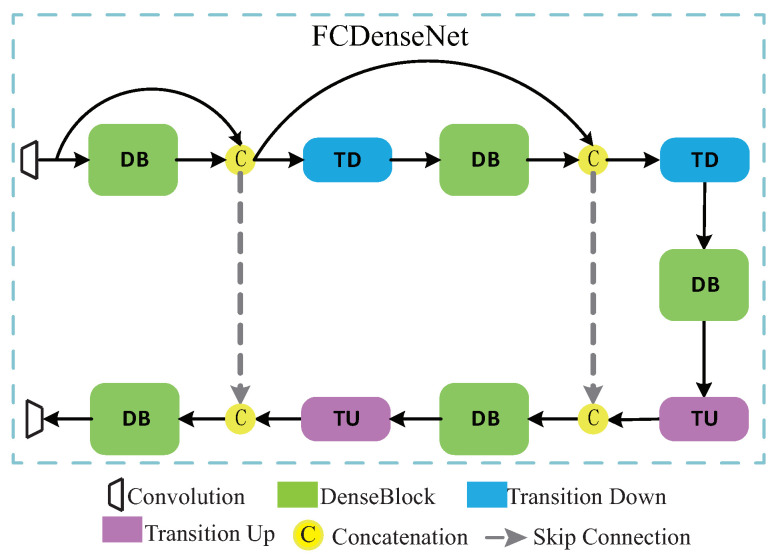
The Structure of FCDenseNet.

**Figure 4:**
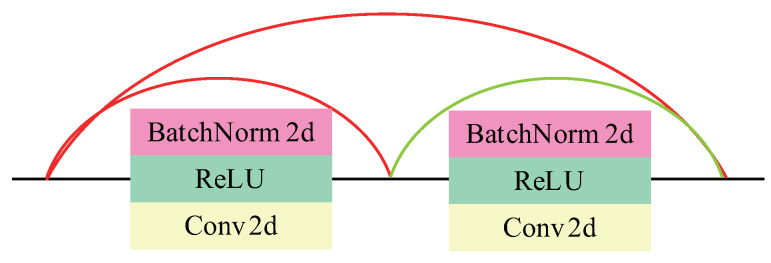
The Structure of two DB Blocks.

Fully Convolutional Densely Connected Network (FCDenseNet) [27] proposed by Jégou et al. in 2016, has achieved excellent results in the segmentation of pictures and videos. The detailed structure diagram in Figure 2d is shown in Figure 3, which is composed of five DBs, two TDs, and two TUs. The representation of DB is shown in Figure 4, and the layers are connected to each other. TD and TU stand for down-sampling and up-sampling, respectively. The purpose of down-sampling in this article is to better integrate the features of the secret image and the carrier image. The purpose of up-sampling is to restore the features of the carrier image as high as possible while retaining the main characteristics of the secret image. TD is composed of BN, ReLU, 1 × 1Conv, and 2 × 2 maximum pooling layers. TU consists of 3 × 3 inverse convolutions with a step size of 2. In order to describe FCDenseNet in more detail, as shown in Table 2, we can see the execution process of the network in detail. Note that we only used 5 DBs.

### 3.2. Decoder for Reconstructing the Secret Image

In the decoder, there are two extraction networks with the same structural design. The input of the decoder is a three-channel color image, and the output is two different secret images. Figure 2a is a brief diagram of the extraction network, and the detailed structure is shown in Table 3. For the convolution operation, all padding and step sizes are 1.

## 4. Experimental Results and Analysis

In this article, the dataset comes from the ImageNet image database. We use 20,000 images as the training set to train the SteganoCNN network. A total of 5000 pictures were used as a test set to evaluate the network. This experiment is divided into three groups: one is ordinary CNN, one is UNet, and the other is FCDenseNet. To facilitate the analysis and comparison of experiments, the three groups of experiments are set to the same parameters, the number of iterations is set to 200, the batch is set to 15, the initial learning rate is 0.0001, and the weight is equal to 0.75. In this experiment, our hardware is set to RTX2070, and the Graphics Processing Unit memory is 8G. The experimental platform environment is Pytorch 1.1.0, Python 3.6.8, and simulation experiments are carried out based on this environment. The final result of the experiment is analyzed through visual and quantitative evaluation, and the safety of steganography is checked by steganalysis tools.

In our three sets of experiments, the memory space occupied by each model and the memory space occupied by the corresponding parameters is shown in Table 4. The memory space occupied by the model includes the memory space occupied by the parameters, that is, excluding the parameters, all three models are 0.1 M. The size of the model is mainly determined by the hidden network. Different hidden networks have different parameters. Therefore, the size of the hidden network parameters determines the size of the entire model. It can be seen from Table 4 that the memory space occupied by FCDenseNet is the smallest.

Table 5 shows the convergence speed and the total loss of iteration for the three sets of experiments. For ordinary CNN, when the number of iterations is 73, the total loss of the model does not change. When the number of iterations of Unet is 184, the loss of the model does not change. When the number of iterations of FCDenseNet is fixed at 109, the total loss of the model does not change. It can be concluded from Table 5 that if the model converges quickly, the total loss is not necessarily the smallest, and if the model converges slowly, the total loss is also not necessarily the smallest. The convergence speed of the model and the loss of the model depend on the internal structure of the model. We can see that the hidden network uses FCDenseNet, and the total loss of the model is minimized.

### 4.1. Subjective Visual Assessment

**Figure 5:**
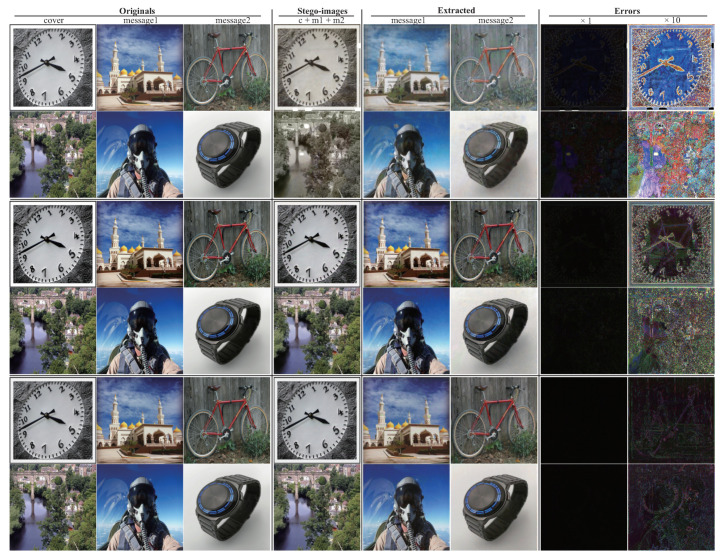
Random samples are from the SteganoCNN network, 1 and 2 rows, 3 and 4 rows, 5 and 6 rows correspond to ordinary CNN, Unet, FCDenseNet samples, respectively.

We introduce a more common human vision evaluation method to evaluate SteganoCNN. It can be seen from Figure 5 that the FCDenseNet used in the hidden network has the smallest error and the best effect. The hidden ability of Unet network is second, and ordinary CNN has the worst effect. Therefore, FCDenseNet or Unet can be selected as our hidden network. After the value of each pixel corresponding to the error map of FCDenseNet is multiplied by 10, we can only see the artifact of a secret image because of the relatively flat area of the carrier image. Although the extraction network of our model is the same, it uses different networks to hide, and the extraction network cannot reconstruct the secret image. In other words, the extraction network corresponding to the trained Unet cannot reconstruct the stego-image generated by the trained FCDenseNet. This is because the parameters of the trained model are different. If an attacker steals a network, the attacker cannot reconstruct the secret image in another network by using the extraction network in this network.

### 4.2. Objective Quantitative Evaluation

#### 4.2.1. Peak Signal-to-Noise Ratio and Structural Similarity

For quantitative evaluation, the Peak Signal to Noise Ratio (PSNR) [28] and the Structural Similarity Index (SSIM) [29] are used to evaluate the performance of SteganoCNN. The larger the PSNR, the smaller the distortion of the image, and the higher the image quality. The closer the value of SSIM is to 1, the smaller the distortion of the image. For two images, the definition of PSNR is as follows

(2) $$MSE=\frac{1}{WH}\sum_{i=1}^{W}\sum_{j=1}^{H}{\left(X_{i,j}-Y_{i,j}\right)}^{2}$$

(3) $$PSNR=10\log\frac{{\left(2^{n}-1\right)}^{2}}{MSE}$$

MSE represents the mean square error between the image *X* and the image *Y*, *W* and *H* represent the width and height of the image, respectively. $2^{n}-1$ is the maximum value of pixels, where *n* is the number of bits per pixel.

SSIM can be used to compare the similarity of two images in structural distribution. For two images and, SSIM can be calculated by the following formula

(4) $$SSIM=\frac{\left(2\mu_{X}\mu_{Y}+k_{1}R\right)\left(2\sigma_{XY}+k_{2}R\right)}{\left(\mu_{X}^{2}+\mu_{Y}^{2}+k_{1}R\right)\left(\sigma_{X}^{2}+\sigma_{Y}^{2}+k_{2}R\right)}$$

where $\mu_{X}$ and $\mu_{Y}$ are the mean value of image *X* and *Y*, respectively; $\sigma_{X}^{2}$ and $\sigma_{Y}^{2}$ are the variance in image *X* and the variance in image *Y*. By default, the value of SSIM is between [−1, 1], where 1.0 means that the two images are the same.

Table 6 corresponds to Figure 5, which respectively counts the PSNR and SSIM of the carrier image and the stego-image, secret image 1, reconstructed secret image 1, secret image 2, and reconstructed secret image 2. Table 6 shows 300 samples randomly selected. The hidden network uses the average PSNR and SSIM corresponding to ordinary CNN, Unet, and FCDenseNet. It can be seen from Table 7 that for the SteganoCNN we designed, the order of hiding effect is FCDenseNet, Unet, and ordinary CNN. Since two pictures are hidden, the corresponding PSNR and SSIM are relatively low, but the FCDenseNet network, carrier image and stego-image, secret image 1 and reconstructed secret image 1, secret image 2 and reconstructed secret image 2 correspond to the PSNR and SSIM reached (35.852, 0.981), (31.695, 0.954), (30.553, 0.948), and the experimental results obtained should be at the upper-middle level.

#### 4.2.2. Relative Capacity and Payload Capacity

To further verify the validity of our experiment, we quantify the experiment with the relative capacity and load rate of the image. Compared with the information-hiding schemes related to deep-learning in recent years, the relative capacity of the scheme we designed is high. The relative capacity is defined as follows

(5) Relative_capacity=Absolute_capacityimage_size

In [30], only one image is hidden, one image is reconstructed, and the number of reveal networks is one. StegoCNN hides two images and reconstructs two images. StegoCNN uses two reveal networks. It can be seen from Table 8 that the relative capacity of our scheme is 2, which is the highest.

Payload capacity is a commonly used evaluation index for the direction of information hiding. The higher the payload capacity, the greater the amount of information carried, but the payload capacity is inversely proportional to the image quality. We ensure that the image payload capacity is relatively large enough while ensuring that the image has a relatively high quality. After calculation, the payload capacity of our proposed SteganoCNN scheme is 47.92 bits per pixel, and the payload capacity is defined as follows

(6) $$r=1-\frac{1}{W\times H}\sum_{i=1}^{W}\sum_{j=1}^{H}\left|s_{i,j}-s{}_{i,j}^{\prime }\right|$$

**Figure 6:**
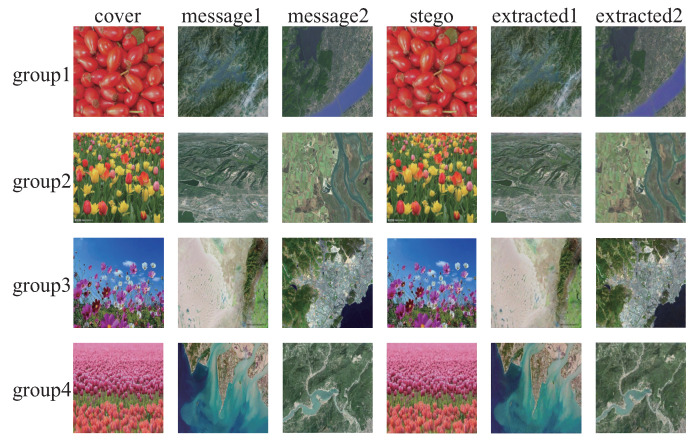
The hiding and extraction of random samples of remote sensing images. The first column is the carrier image, the second and third columns are the original secret image, the fourth column is the stego-image, and the fifth and sixth columns are the extracted secrets image.

(7) payloadcapacity=r×8×3(bpp)

where 8 means that a single pixel occupies eight bits, and 3 means that each image has three channels. That is the average number of secret information bits hidden in each pixel of the carrier image. For Figure 6, we report the payload capacity of the stego-image in Table 9.

### 4.3. Generalization Ability

**Figure 7:**
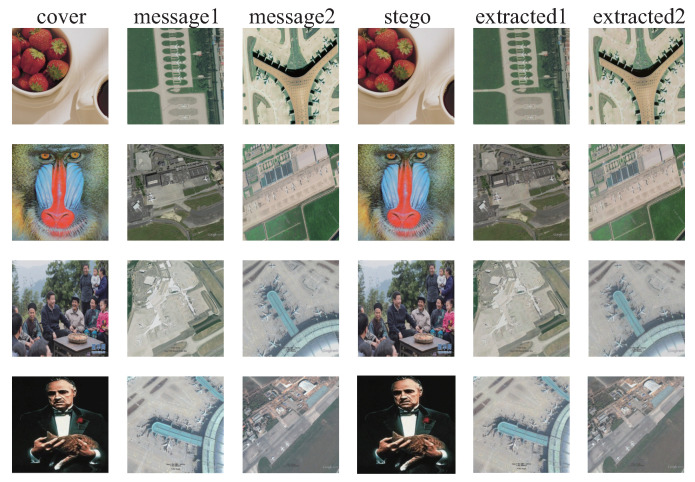
The hiding and extraction of random samples of UAV aerial images. The first column is the carrier image, the second and third columns are the original secret images, the fourth column is stego-image, and the fifth and sixth columns are the extracted secret image.

**Figure 8:**
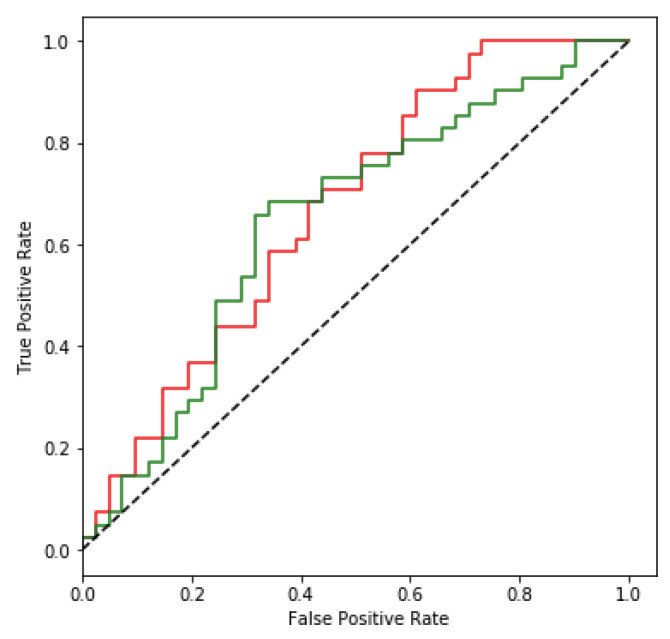
The red and green curves correspond to the ROC curves of FCDenseNet in ImageNet, remote sensing data, and aerial data, respectively. The red curve and the green curve use the same number of images, the number is 400. Among them, there are 50 carrier images and 350 stego-images.

The generalization ability of the SteganoCNN model, that is, the test on the non-ImageNet dataset, can also achieve good results. To verify the generalization ability of our model and the anti-steganalysis ability of the data after generalization, we used remote-sensing images and images taken by an Unmanned Aerial Vehicle (UAV) as secret images to hide. The hidden network uses FCDenseNet, in which the experimental results of the remote sensing image are shown in Figure 6, and the image taken by the UAV is shown in Figure 7. In addition, we also tested CT images, cell images, etc. The final results meet the requirements of steganographic security. This further verifies that we have a good generalization ability. Figure 8 shows that our model also has a certain degree of anti-steganalysis ability on remote-sensing images and aerial images.

### 4.4. Steganalysis

We use the StegExpose [36] least significant bit detection tool to analyze the data we tested, using a standard threshold of 0.2. StegExpose is composed of four analysis methods: sample pair analysis, Chi-Square analysis, RS analysis, and Primary set analysis. Figure 8 shows the analysis result of the StegExpose tool. The horizontal axis: False Positive Rate indicates that no embedded message is judged to be an embedded message, and the vertical axis: True Positive Rate indicates that the embedded message is judged to be an embedded message. The black diagonal line represents random guessing, and the information hiding scheme we designed is almost close to random guessing through the analysis of the StegExpose tool. In short, it is impossible to tell whether the image is embedded in the message, indicating that our scheme has a certain degree of anti-steganalysis ability.

## 5. Conclusions

This paper proposes a new SteganoCNN scheme based on deep learning. This paper realizes to hide two secret images into one carrier image. The sender obtains the stego-image and transmits it to the receiver by concatenation of the three images into the encoder, and the receiver receives the stego-image from the decoder through the decoder. Two required secret images are reconstructed. Besides this, the trained StegoCNN is suitable for almost all color image steganography, including remote-sensing images and aerial images. In the next step, we will try to prune our entire model to further reduce the number of model parameters and achieve faster hiding and extraction. Besides, we are ready to introduce this solution to the mobile terminal to truly realize the concept of combining technology with practical applications.

## Figures and Tables

**Table 1:** Common CNN network structure.

Process	Output Size
Reflectionpad2d + 7×7Conv+ InstanceNorm2d + ReLU	256×256×64
3×3Conv + InstanceNorm2d + ReLU	128×128×128
3×3Conv + InstanceNorm2d + ReLU	64×64×128
2(Reflectionpad2d + 3×3Conv+ InstanceNorm2d + ReLU)	64×64×256
2(Reflectionpad2d + 3×3Conv+ InstanceNorm2d + ReLU)	64×64×256
2(Reflectionpad2d + 3×3Conv+ InstanceNorm2d + ReLU)	64×64×256
3 × 3ConvTranspose2d + InstanceNorm2d + ReLU	128×128×128
3 × 3ConvTranspose2d + InstanceNorm2d + ReLU	256×256×64
Reflectionpad2d + 7×7Conv+ Tanh()	256×256×3

**Table 2:** FCDenseNet network structure.

Process	Output Size
3 × 3*Conv*	256×256×50
2(BN + ReLU + 3 × 3*Conv*)	256×256×150
TD + 2(BN + ReLU + 3 × 3*Conv*)	256×256×250
TD + 2(BN + ReLU + 3 × 3*Conv*) + TU	256×256×350
2(BN + ReLU + 3 × 3*Conv*) +TU	256×256×250
2(BN + ReLU + 3 × 3*Conv*)	256×256×350
1×1Conv	256×256×3

**Table 3:** Reveal Network Structure.

Process	(Padding, Stride)	Output Sizse
3×3Conv + BN + ReLU	(1, 1)	256×256×64
3×3Conv + BN + ReLU	(1, 1)	256×256×128
3×3Conv + BN + ReLU	(1, 1)	256×256×256
3×3Conv + BN + ReLU	(1, 1)	256×256×128
3×3Conv + BN + ReLU	(1, 1)	256×256×64
3×3Conv + BN + Sigmoid	(1, 1)	256×256×3

**Table 4:** Comparison of model size and parameter size corresponding to different hidden networks.

Hiding Network	Model Size (M)	Parameter Size (M)
CNN	22.2	22.1
Unet	165.1	165
FCDenseNet	10.9	10.8

**Table 5:** Convergence speed of different hidden networks and total loss of iterations.

Hiding Network	Number of Iterations	Total Model Loss
CNN	73	0.040715
Unet	184	0.003957
FCDenseNet	109	0.002379

**Table 6:** PSNR and SSIM corresponding to ordinary CNN, Unet, FCDenseNet.

	Carrier	Reconstructed	Reconstructed
Figure	vs. Stego-Image	s1’ vs. s1	s2’ vs. s2
	(PSNR, SSIM)	(PSNR, SSIM)	(PSNR, SSIM)
Figure 5, row1	21.614, 0.851	23.916, 0.855	19.532, 0.728
Figure 5, row2	22.572, 0.821	25.298, 0.881	26.535, 0.971
Figure 5, row3	28.739, 0.946	30.018, 0.943	27.668, 0.909
Figure 5, row4	29.044, 0.932	32.179, 0.947	31.926, 0.949
Figure 5, row5	39.004, 0.986	30.418, 0.954	29.873, 0.937
Figure 5, row6	36.322, 0.995	29.189, 0.948	30.149, 0.970

**Table 7:** Average PSNR and SSIM corresponding to ordinary CNN, Unet, FCDenseNet.

	Carrier	Reconstructed	Reconstructed
Models	vs. Stego-Image	s1’ vs. s1	s2’ vs. s2
	(PSNR, SSIM)	(PSNR, SSIM)	(PSNR, SSIM)
CNN	20.274, 0.861	22.932, 0.859	22.271, 0.831
Unet	29.554, 0.921	30.540, 0.913	28.963, 0.928
FCDenseNet	35.852, 0.981	31.695, 0.954	30.553, 0.948

**Table 8:** Average PSNR and SSIM corresponding to ordinary CNN, Unet, FCDenseNet.

Schemes	Absolute Capacity	Stego-Image Size	Relative Capacity
	(Bytes/Image)		(Bytes/Pixel)
[18]	1×224×224	224×224	1
[31]	≥37.5	64×64	9.16 × 10${}^{-3}$
[32]	1.125	512×512	4.29 × 10${}^{6}$
[33]	3.72	≥512×512	1.42 × 10${}^{-5}$
[34]	1533∼4300	1024×1024	1.46 × 10${}^{-3}$∼4.10 × 10${}^{-3}$
[35]	0.375	32×32	3.7 × 10${}^{-4}$
[30]	1×256×256	256×256	1
Ours	2×256×256	256×256	2

**Table 9:** Payload capacity of randomly selected samples.

Figure 6	Payload Capacity (BPP)
group1	47.928
group2	47.924
group3	47.922
group4	47.928
Average	47.925
